# RNAi-derived transgenic resistance to Mungbean yellow mosaic India virus in cowpea

**DOI:** 10.1371/journal.pone.0186786

**Published:** 2017-10-27

**Authors:** Sanjeev Kumar, Bhaben Tanti, Basavaprabhu L. Patil, Sunil Kumar Mukherjee, Lingaraj Sahoo

**Affiliations:** 1 Department of Bioscience and Bioengineering, Indian Institute of Technology Guwahati, Guwahati, India; 2 Department of Botany, Gauhati University, Guwahati, Assam, India; 3 ICAR-National Research Centre on Plant Biotechnology, LBS Centre, IARI, Pusa Campus, New Delhi, India; 4 Division of Plant Pathology, Indian Agricultural Research Institute, New Delhi, India; Shanghai Institutes for Biological Sciences, CHINA

## Abstract

Cowpea is an important grain legume crop of Africa, Latin America, and Southeast Asia. Leaf curl and golden mosaic diseases caused by Mungbean yellow mosaic India virus (MYMIV) have emerged as most devastating viral diseases of cowpea in Southeast Asia. In this study, we employed RNA interference (RNAi) strategy to control cowpea-infecting MYMIV. For this, we generated transgenic cowpea plants harbouring three different intron hairpin RNAi constructs, containing the *AC2*, *AC4* and fusion of *AC2* and *AC4* (*AC2+AC4*) of seven cowpea-infecting begomoviruses. The T_0_ and T_1_ transgenic cowpea lines of all the three constructs accumulated transgene-specific siRNAs. Transgenic plants were further assayed up to T_1_ generations, for resistance to MYMIV using agro-infectious clones. Nearly 100% resistance against MYMIV infection was observed in transgenic lines, expressing *AC2-*hp and *AC2*+*AC4-hp* RNA, when compared with untransformed controls and plants transformed with empty vectors, which developed severe viral disease symptoms within 3 weeks. The *AC4-hp* RNA expressing lines displayed appearance of milder symptoms after 5 weeks of MYMIV-inoculation. Northern blots revealed a positive correlation between the level of transgene-specific siRNAs accumulation and virus resistance. The MYMIV-resistant transgenic lines accumulated nearly zero or very low titres of viral DNA. The transgenic cowpea plants had normal phenotype with no yield penalty in greenhouse conditions. This is the first demonstration of RNAi-derived resistance to MYMIV in cowpea.

## Introduction

Cowpea (*Vigna unguiculata* L. Walp.) is one of the most important warm-season food and forage legumes cultivated across sub-Saharan Africa, Central and South America, Europe, Southeast Asia, and the United States [1–2]. Cowpea grains and green peas provide a valuable revenue-source for resource poor farmers of the developing world [3–4]. It is an important source of nutrition due to high protein content, palatability, and relative freedom from anti-metabolites [5]. Cowpea production suffers losses due to virus infection ranging from 10 to 100% [5]. More than 140 viruses are reported in cowpea, of which 20 viruses are known to have widespread distribution [6–7] and their infection often results in severe yield losses [8]. In sub-Saharan Africa, cowpea chlorotic mottle virus (CPCMV), cowpea severe mosaic virus (CPSMV), cucumber mosaic virus (CMV), cowpea mosaic virus (CPMV), cowpea mild mottle virus (CPMMV), cowpea aphid-borne mosaic virus (CABMV), and cowpea chlorotic mosaic virus (CCMV) are most prevalent in cowpea [9]. In India, cowpea is severely affected by golden mosaic disease (CGMD), and severe leaf curl diseases caused by different isolates of Mungbean yellow mosaic India virus (MYMIV) [10–14]. Yield loss due to viral diseases in legumes including cowpea accounts for approximate $300 million per year [15].

MYMIV belongs to the family *Geminiviridae* and genus *Begomovirus*, which are transmitted by whitefly (*Bemisia tabaci*). Their genome consist of two circular single-stranded DNA components (bipartite, ~2.7 Kb), referred as DNA-A and DNA-B. The DNA-A component encompasses seven ORFs, coding for *AC1*, *AC2*, *AC3*, *AC4* and *AC5* on the complementary strand and *AV1* and *AV2* on the virion strand, which are required for replication and encapsidation. The DNA-B component composed of 2 ORFs, *BC1* and *BV1* which are essential for inter- and intra-cellular movement of the viral genome respectively in the host [16–17]. There are no known natural sources of resistance to MYMIV in cowpea, and hence, resistance breeding is difficult to achieve. RNA-interference (RNAi) strategy has emerged as an efficient means to control begomoviruses infection in crops including legumes [18–23]. RNA silencing also called as Post-Transcriptional Gene Silencing (PTGS), in which the degradation of target RNA occurs in a sequence-specific manner via formation of double-stranded RNA, that are processed into small interfering RNAs (siRNA) by the Dicer-Like (DCL) proteins and the RNA-Induced Silencing Complex (RISC) [24–29]. RNAi-derived transgenic resistance has been accomplished by targeting the *AC1* of different geminiviruses, including Tomato yellow leaf curl virus (TYLCV) [19], Bean golden mosaic virus (BGMV) [30], African cassava mosaic virus (ACMV) [31–32], and Maize streak virus (MSV) [33], whereas targeting the common/intergenic region has resulted in complete arrest of MYMV [34] and ACMV [35].

The transcriptional activator protein AC2 (TrAP), a multifunctional protein encoded by both monopartite as well as bipartite begomoviruses, is known to activate the viral late gene promoters [36–37], suppress gene silencing [38–40], and determine pathogenicity. Use of RNAi strategy to silence *AC2* has been effective in controlling geminivirus infection in tobacco [22, 41].

RNAi targeting of *AC4*, an important geminivirus gene embedded within *AC1* ORF, has resulted in resistance to in cassava-infecting geminiviruses through silencing suppression activity [42]. These observations indicate both *AC2* and *AC4* are potentially important RNAi targets for controlling geminiviruses. In this study, we generated transgenic cowpea expressing hpRNA of RNAi suppressors, *AC2* and *AC4*, and evaluated these lines for resistance to MYMIV. We present for the first time, the RNAi-mediated resistance in cowpea against MYMIV.

## Material and methods

### Survey for virus isolates and sequence analysis

We surveyed the cowpea crops for yellow mosaic symptoms at the vegetative growth stage in five states of India, namely, Jharkhand, Chhattisgarh, Uttar Pradesh, Madhya Pradesh and Assam, during 2012–13 [43–44]. The maximum incidence of this viral disease was in a field of Jharkhand state, with a disease incidence of about 60–70%. The cowpea plants in the field exhibited stunted growth, yellow patches, mottling of leaves, reduced leaf size and distortion of leaf lamina symptoms (S1 Fig). The infected plant leaf materials were RCA analyzed, cloned and sequenced. Agroinfectious dimeric clones were prepared for MYMIV cowpea isolate, propagated and maintained in cowpea cultivar Pusa Komal through agroinfiltration and maintained in a greenhouse at 25–27°C. Total genomic DNA was extracted from infected leaf samples by using DNA isolation kit (Hi-Media, Mumbai), the purified genomic DNA were subjected to amplify full length of DNA-A and DNA-B, using TempliPhi^™^ DNA-Amplification kit (GE Healthcare, UK) through Rolling circle amplification (RCA) method as per manufacturer’s instructions. The ~2.7 Kb DNA fragments obtained after the restriction digestion (*Eco*RI, *Hind*III, *Bam*HI, *Sac*I and *Eco*RV) of the RCA products were cloned and sequenced. The sequences obtained were analyzed using DNA Star, Mega 5.2, and BIOEDIT version 7.0 programs.

### RNAi vector construction

**Fig 1:**
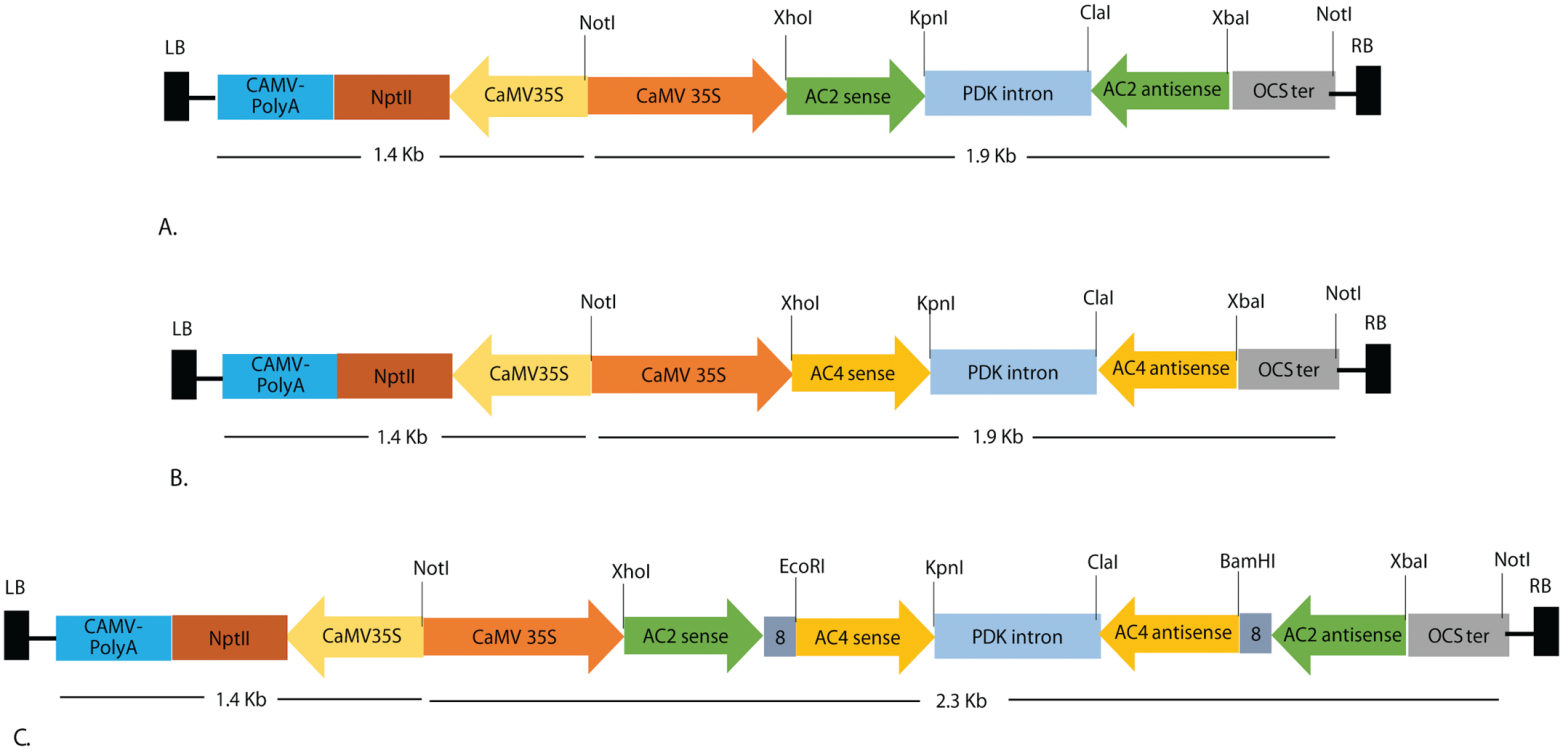
Schematic T-DNA map of pART27 RNAi cassettes of MYMIV-AC2, MYMIV-AC4 and MYMIV AC2+AC4 stack in sense and antisense orientation. Abbreviations: LB, left border; RB, right border; CaMV 35SP: Cauliflower mosaic virus 35S promoter; OCS terminator: octopine synthase terminator, PDK intron: pyruvate dehydrogenase kinase intron, CaMV 35S poly-A, Cauliflower mosaic virus 35S terminator; NPTII: Neomycin phosphotransferase; Restriction enzyme NotI were used for cloning of all the three RNAi cassettes from the intermediate RNAi vector pKANNIBAL into the plant transformation binary vector pART27.

Three hairpin constructs targeting conserved regions of *AC2*, *AC4* and fusion (*AC2*+*AC4* stack) ORFs of seven cowpea infecting begomoviruses (S1 Table) were made. The 186 nt fragment of *AC2* and 197 nt fragment of *AC4* ORFs were amplified by polymerase chain reaction (PCR) from the DNA-A genome of MYMIV cowpea isolate. The fragments were cloned in sense orientation, at the restriction sites *Xho*I and *Kpn*I, and in antisense orientation, at the restriction sites *Xba*I and *Cla*I, on either sides of Pdk-intron of the intermediate vector, pKANNIBAL (CSIRO, Plant Industry, Canberra, Australia). For construction of *AC2*+*AC4* stack RNAi construct, the sense fragments of *AC2* (*Xho*I and *Eco*RI) and *AC4* (*Eco*RI and *Kpn*I) interrupted by 8 nt gap, and antisense fragments of *AC2* (*Xba*I and *Bam*HI) and *AC4* (*Bam*HI and *Cla*I) interrupted by 8 nt gaps (Fig 1) were cloned on either side of the Pdk intron of pKANNIBAL (Primer details given in S2 and S3 Tables). For generating stable transgenic plant lines, the RNAi cassettes under the control of CaMV35S promoter and OCS terminator (as *Not*I fragments) were subcloned into the plant binary vector, pART27 (CSIRO, Plant Industry, Canberra, Australia).

### Construction of agroinfectious dimeric clones

To check the virus infectivity of MYMIV in the host plant, we constructed the *Agroinfectious* dimeric clones of MYMIV DNA-A [KY556679] and DNA-B [KY556680] by using a high-fidelity PCR-based strategy. Two different sets of primers specific for the amplification of MYMIV, complete DNA-A and DNA-B were designed. For amplification of DNA-A, forward primer 5’-**GAATTC** ATG GGCGCGCAAAG-3’ and the reverse primer 5’-**TCTAGA**TTCAATAATGTGGATCAACG-3’ were synthesized commercially. The underlined nucleotides indicate *Eco*RI restriction site in the forward primer and *Xba*I in the reverse primer. The PCR conditions used were as follows, denaturation at 95°C for 3 min, 95°C for 1 min, annealing 60°C for 1 min and extension for 3 min at 72°C for 30 cycles followed by a final extension at 72°C for 10 min. The amplified PCR products ~2.6–2.7 Kb were gel purified using Hi-Yield^™^ Gel/PCR DNA Mini Kit (Hi-Media, Mumbai, India) and subsequently cloned into pTZ57R/T vector (Thermo Scientific, USA) and the clones were confirmed by PCR, restriction digestion, and sequencing. The *Eco*RI-*Xba*I (2.7 kb) fragment from the recombinant pTZ57R/T clone of DNA-A was subcloned in a plant binary vector pCAMBIA3300 as *Eco*RI-*Xba*I insert resulting in pC-A’. Subsequently, the 2.7 kb *Eco*RI viral DNA fragment from pUC18-DNA-Awas recloned in pC-A’ to generate a complete DNA-A dimer in tandem in pCAMBIA3300 (named as pC-2.0A). The orientation of the dimeric construct of DNA-A was confirmed by *Mfe*I (unique cutter), *Dra*I (unique cutter) for DNA-B present in viral DNA sequence.

The *Agroinfectious* dimeric construct of MYMIV DNA-B was also prepared in a similar fashion. Two different set of primers were designed: 5’-**AAGCTT**TTATAGGACATTTGCT-3’ and reverse primer 5’-**GAATTC** AAGCTTTGTAAAGCAATG-3’. The underlined nucleotides represent *Eco*RI restriction site tagged to the forward primer and *Hind*III restriction site tagged to the reverse primer. The PCR conditions were same as described for DNA-A with annealing temperature changed to 58°C for 1 min. The PCR products were gel purified and subsequently cloned into pTZ57R/T vector (Thermo Scientific, USA) and the clones were confirmed by PCR, restriction digestion, and sequencing. The *Eco*RI-*Hind*III fragment (2.6 kb) digested from the pTZ57R/T DNA-B vector was subsequently cloned in a plant binary vector pCAMBIA3300 resulting in pC-B’. The 2.6 kb *Hind*III viral DNA fragment from pUC18-DNA-B was recloned in pC3300-A’ at *Hind*III site to generate the dimeric clone of DNA-B (pC3300-2.0B). The insert orientation was confirmed by restriction digestion with unique cutter *Dra*I that had internal site in the MYMIV DNA-B genome.

Both the dimeric clones, pC3300-2.0A and pC3300-2.0B were mobilized into *Agrobacterium tumefaciens* strain EHA105 by triparental mating using helper plasmid pRK2013. *Agrobacterium* trans-conjugates were confirmed by colony PCR using the primers specific to internal regions of DNA-A and DNA-B. The empty vector pCAMBIA3300 mobilized to *Agrobacterium* served as negative control for mock inoculation in control plants [44].

### Agroinfiltration

The agroinfectious clones of MYMIV DNA-A and DNA-B were cultured separately in YEP medium containing antibiotics (20 mg/L rifampicin and 50 mg/L kanamycin) and grown overnight at 28°C to reach an OD_600_ = 0.6. Cells were then harvested and resuspended in an equal volume of 10 mM 2-(*N-* morpholino) ethanesulfonic acid (MES) buffer and 10 mM MgCl_2_, pH 5.6 and 200 μM acetosyringone. The resuspended cells were kept for shaking at 90 rpm at 28°C for 1 h, and subsequently used to infiltrate the abaxial surface of young trifoliate leaves of four weeks-old WT and RNAi-transgenic cowpea plants. The MYMIV inoculated cowpea plants were maintained in a greenhouse.

### Production of transgenic cowpea plants and its molecular characterization

**Fig 2:**
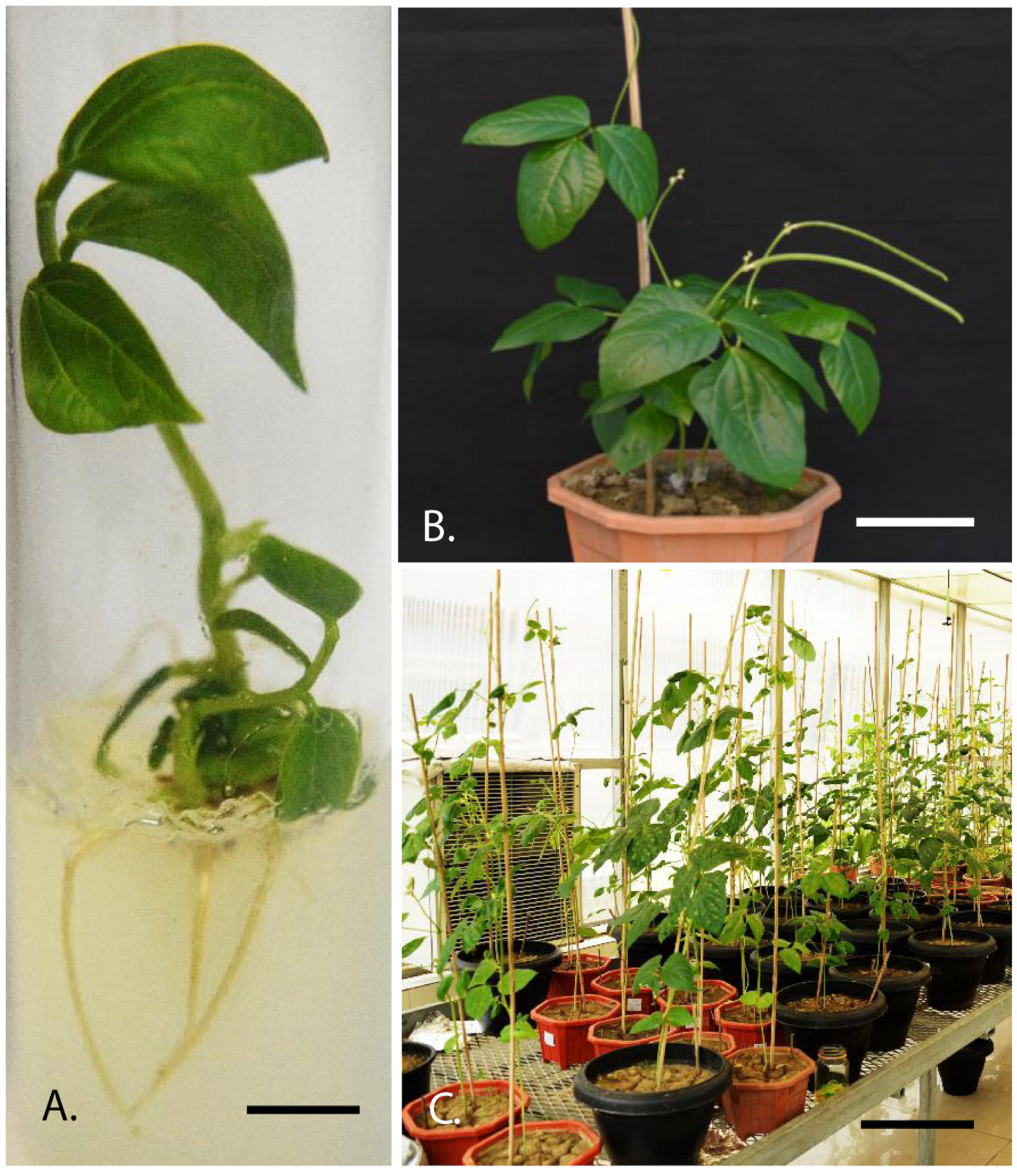
Generation of transgenic cowpea (*Vigna unguiculata)* cv. PUSA Komal through *Agrobacterium* mediated transformation. **A.** Transformed shoot on rooting medium (bar 2 cm), **B.** Putative transformed plant established in soil (bar 10 cm), **C.** T_1_ and T_2_ cowpea progeny plants growing in transgenic greenhouse containment.

Cowpea were transformed using all the three RNAi constructs (pART27-MYMIV-*AC2*, pART27-MYMIV-*AC4*, and pART27-MYMIV-*AC2*+*AC4*) through *Agrobacterium*-mediated transformation of cotyledonary node explants established in our lab [45], and transgenic plants were established in greenhouse at 25±2°C. T_0_ plants were screened by PCR and the PCR-positive lines were advanced to T_1_ and T_2_ generations [45] and homozygous lines were selected (Fig 2).

Genomic DNA was isolated from non-transformed (WT) and transgenic plants (T_0_, T_1_and T_2_) using the DNA isolation kit (Hi-Media, Mumbai, India). PCR was performed in a thermal cycler (BioRad, USA) to detect the presence of *npt*II, MYMIV-*AC2* and MYMIV-*AC4* using primers specific to these transgenes (S2 and S3 Tables). The PCR products were analyzed under UV light after electrophoresis on a 1% agarose gel and staining with ethidiumbromide.

For Southern hybridization, the genomic DNA was isolated from the WT and T_0_ PCR positive plants using NucleoSpin Plant II Maxi (Takarabio, Clontech, Japan) and 60 μg of genomic DNA from each plant was digested with *Eco*RI (Thermo Fisher Scientific, USA) and resolved on 0.8% agarose gel. The completely digested and purified DNA was blotted onto Zeta-Probe membrane (Bio-Rad, Hercules, CA, USA). The blot was hybridized with the DIG-labeled 0.54 kb PCR product corresponding to the coding region of *npt*II. Southern hybridization was performed according to the manufacturer’s instructions of the DIG Labeling and Detection kit (Roche Diagnostics, Mannheim, Germany).

The plants in T_1_ and T_2_ generation were checked for transgene segregation by PCR as described in previous section. The independent transgenic events segregating transgenes to all its offsprings (in the T_1_ and T_2_ generations) were considered as homozygous [46].

### Plant inoculation with MYMIV and symptom evaluation

Four weeks old T_0_ and T_1_ transgenic and WT cowpea plants were inoculated with MYMIV agroinfectious dimers (of DNA-A and DNA-B) under the greenhouse conditions. Inoculated plants were maintained in the greenhouse for symptom development and evaluation. Five T_0_ transgenic lines of each constructs, five T_1_ plants from each transgenic line, plants transformed with empty vector pART27 and WT plants were used for each experiment. Symptom evaluations were carried out every alternate day for a period of 10 weeks or until development of complete disease symptoms on control plants. The symptomatic plants were photographed and processed for molecular analysis through RCA, qRT-PCR and Northern hybridization. Disease symptoms were scored using a scale of 0–5 [47] and resistance levels were determined for each plant line. Plants transformed with the empty vector pCAMBIA3300 (used for agroinfectious dimer preparation) served as negative controls.

### RT-PCR for detection of MYMIV precoat protein in transgenic plants

Total RNA was extracted using a NucleoSpin RNA Plant Kit (Takara, Clontech, Japan) from transgenic plants challenged with MYMIV infectious clones and subjected to RT-PCR (RevertAid^™^ H Minus first-strand cDNA synthesis, Fermentas, USA) using primers (S2 Table) amplifying the 220 bp of *AV2* genes). Amplification of constitutively expressed cowpea ubiquitin (S2 Table) served as a control to check the quality of cDNA synthesized in the RT-PCR (S3 Table).

### Analyses of plants for siRNA accumulation

Transgenic cowpea lines, together with controls, were analyzed for siRNA accumulation by Northern blot hybridization, both before and after challenge with MYMIV. Total RNA was isolated from leaves using TRIZOL Reagent (Invitrogen, CA, USA). Fifty micrograms of RNA was fractionated on a 15% PAGE with 7 M urea and 1× Tris-borate-EDTA (TBE), electroblotted to a Hybond-N^+^ membrane (Amersham Pharmacia Biotech, Buckinghamshire, U.K.), and subjected to Northern blot hybridization with a probe specific to either *AC2* or *AC4* using DIG High Prime DNA labeling and detection kit (Roche Applied Science, Germany). The membranes were processed and the signal from siRNA was detected using CDP-star (Roche Applied Science, Germany), as described in the DIG system and the DIG application manual. The probes were obtained by cloning MYMIV-*AC2* (186 bp) and MYMIV-*AC4* (197 bp) regions in the pGEM-T-Easy vector (and named as pGEM-T/*AC2* and pGEM-T/*AC4*). The cloned segments were subjected to *in vitro* transcription with T7 RNA polymerase for sense strand and by SP6 RNA polymerase for anti-sense strand using the DIG RNA labeling kit (Roche Applied Science, Indianapolis, USA). The DIG-labeled transcript of *AC2* and *AC4* [mixture of sense (T7) and anti-sense (SP6)] were hydrolyzed and denatured at 100°C for 5 minutes and then added to a fresh aliquot of DIG Easy-hyb buffer for hybridization of the membranes for 18 hrs at 42°C [48–50]. The hybridized blots were processed for post-hybridization wash at 42°C and the chemiluminescence based signals were detected using CDP-star as described in the DIG System and the DIG Application Manual (Roche Applied Science, Indianapolis, USA).

### Rolling circle amplification (RCA)

RCA was performed according to the manufacturer instructions of Templiphi 100 amplification kit (GE Healthcare Life Sciences, Pittsburgh, USA) using 100 ng of purified genomic DNA from the virus-challenged cowpea plants. The RCA amplified products were digested with *Mfe*I, unique restriction site present in the genome of MYMIV DNA-A and *Dra*I, present in the genome of DNA-B and the digested products were resolved on 1% agarose gel.

### Viral DNA detection

The amount of viral DNA accumulated in the uppermost leaf of infected plants was estimated by both semi-quantitative and Real-time PCR, 35 days after inoculation. The precoat protein (*AV2*) specific primers (S2 Table) were used to amplify 220 bp internal fragment of *AV2* for detection of viral DNA accumulation (S3 Table). A pair of housekeeping cowpea ubiquitin primers (S2 and S3 Tables) was also used as a control to check the quality of cDNA synthesized in the RT-PCRs.

Real time PCR was performed with the *AV2* specific primers as used in semi-quantitative PCR with cowpea ubiquitin as an internal control, using USB VeriQuest SYBR Green qPCR Master Mix (2X) (Affymetrix, USA) on a Rotor-Gene Q Real-Time PCR System (Qiagen, Germany). The experiment was repeated twice independently with three replicates each. The standard curve was calculated for each sample relative to the expression values. The relative expression of MYMIV *AV2* in WT and transgenic cowpea lines was estimated by normalizing expression values of MYMIV*AV2* with that of housekeeping cowpea-ubiquitin.

### Analysis of agronomic traits of MYMIV-resistant transgenic cowpea lines

The agronomic traits such as plant height, branch number, pod number/plant, seed number/plant, seed weight/plant, 100 seed weight and 10 seed length of the MYMIV resistant T_1_ transgenic cowpea lines were analyzed under greenhouse conditions, and the same were compared with WT plants (S1 Table).

## Results

### Analysis of transgenic cowpea plant lines derived from RNAi-*AC2*, *AC4*, and *AC2*+*AC4* stack

**Fig 3:**
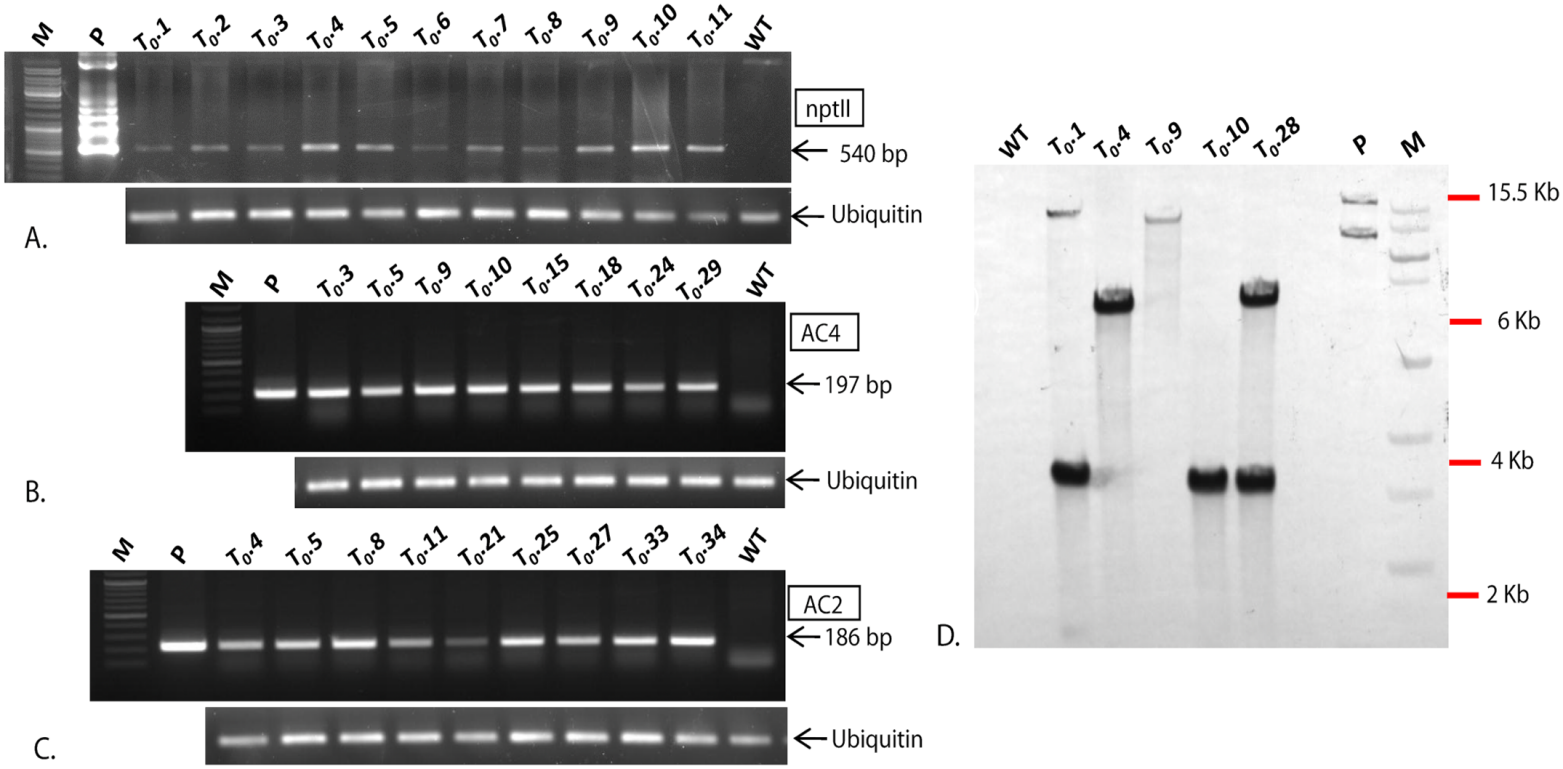
Molecular analysis of transgenic lines overexpressing MYMIV-AC2, MYMIV-AC4 and MYMIV AC2+AC4 stack RNAi constructs. **A-C.** Analysis of kanamycin (*npt*II) resistant T_0_ transgenic cowpea plant lines; **A.** PCR amplification of 540 bp with *nptII* specific primer, **B.** 197 bp amplicon with AC4 gene specific primer, **C.** 186 bp amplicon with AC2 gene specific primer, Polymerase chain reaction (PCR) amplification with Ubiquitin specific primers is shown below for normalization (Fig. A-C.), **D.** Southern blot analysis of 5 independent *Eco*RI digested T_0_ transgenic MYMIV AC2+AC4stackcowpea lines, using *npt*II as probe.

**Fig 4:**
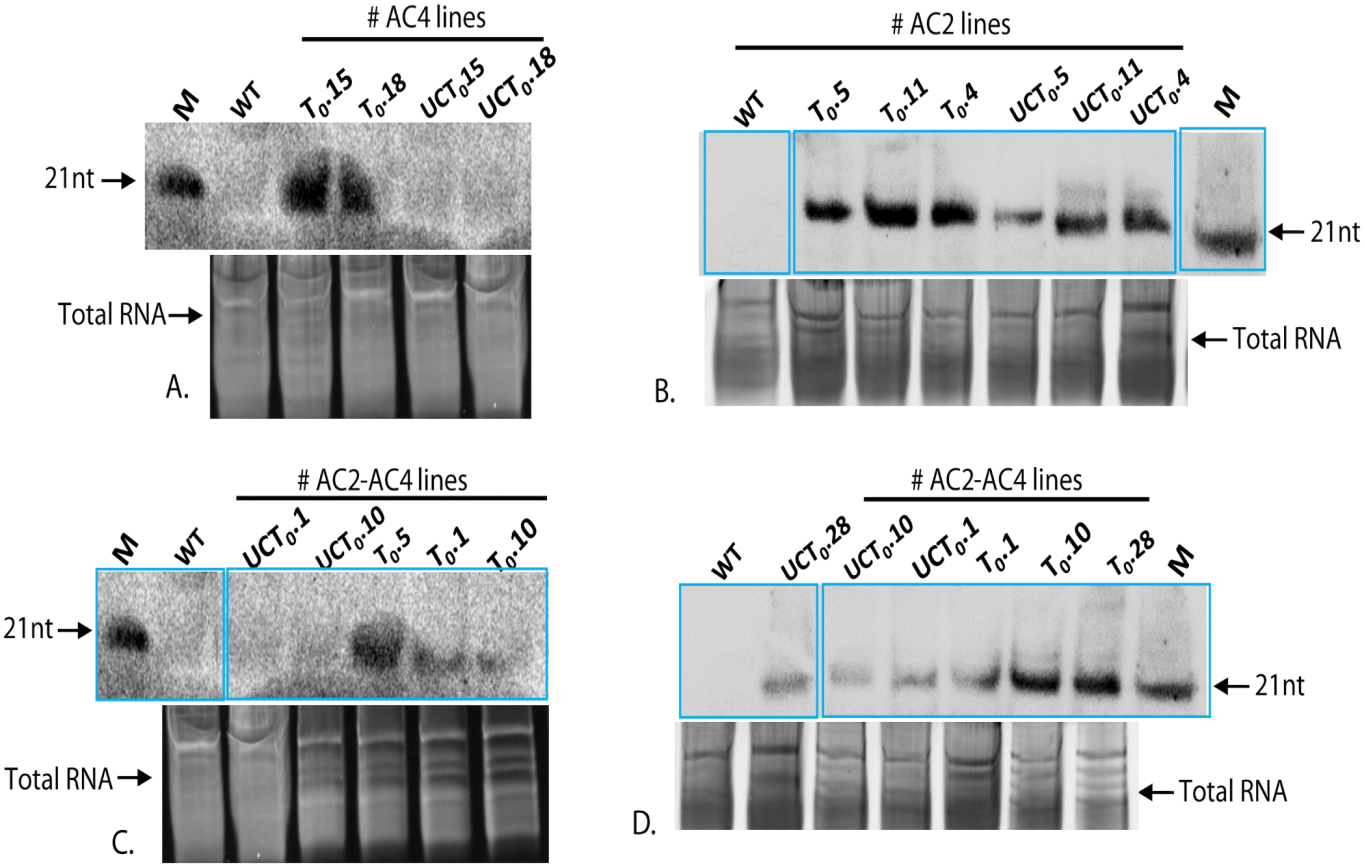
Northern blot analysis of cowpea transgenic lines overexpressing MYMIV-AC4, AC2 and AC2+AC4stacked RNAi constructs for the analysis of siRNA prior and after challenge with MYMIV. Samples are indicated on the top of each lane (UC-Unchallenged). Lower panel shows the total RNA as a loading control. **A.** siRNA accumulation in AC4 overexpression lines; **B.** AC2 overexpression lines and **C-D.** AC2-AC4 stacked overexpression lines. AC4-197nt transcript was used as a probe for hybridization of MYMIV-AC4 and MYMIVAC2+AC4 overexpressing plants. AC2-186nt transcript was used as a probe for hybridization of MYMIV-AC2 and MYMIVAC2+AC4 overexpressing plants, 21nt indicated the siRNA markers.

Twenty-seven putative transgenic T_0_ lines derived from RNAi-*AC2* construct, 34 lines from RNAi-*AC4* construct, and 36 lines from RNAi-*AC2*+*AC4* stacked construct (Figs 1 and 2; Tables 1 and 2) were confirmed for presence of transgenes by PCR (Fig 3A–3C). Randomly chosen five PCR-positive transgenic plant lines generated from RNAi-*AC2*, RNAi-*AC4* and RNAi-*AC2*+*AC4* stacked constructs were analyzed for T-DNA copy number by Southern hybridization using *npt*II probe. The results revealed that three PCR-positive transgenic lines showed transgene integration at varying loci of which 2 transgenic lines (T_0._1 and T_0._10) had integration at the same locus (Fig 3D). Single-copy insertion was detected in the line T_0_.1, whereas the additional mild signal corresponding to 13 kb appeared in line T_0_.1 possibly resulted due to the hybridization of the probe to unprocessed cowpea genomic DNA (Fig 3D). Single-copy insertion was also detected in line T_0_.4 and T_0_.10 whereas T-DNA insertion was absent in the line T_0_.9, a faint signal corresponding to >13 kb was possibly due to hybridization of the probe to unprocessed genomic DNA, and two copies were detected in the transgenic line T_0_.28 (Fig 3D). In the case of RNAi-*AC2*, line T_0._5 and T_0_.11 showed single copy while line T_0_.4 had double copy, similarly in case of RNAi-*AC4*, line T_0_.15 detected with single copy, line T_0_.15 and T_0_.18 showed two copies (Data not shown). The PCR-positive T_0_ transgenic lines from all the three RNAi constructs were further evaluated for the accumulation of *AC2* and *AC4* specific siRNA by Northern blotting. The data presented was for three RNAi-*AC2 lines* (Fig 4B), two RNAi-*AC4* lines (Fig 4A), and four RNAi-*AC2*+*AC4* stacked lines (Fig 4C and 4D). All the RNAi lines showed accumulation of transgene-specific siRNAs (Fig 4A–4D). However, level of siRNA accumulation was highest in *AC2* line #T_0_.11, followed by *AC2* lines #T_0_.5 and #T_0_.4 (Fig 4B), *AC4* lines #T_0_.15 and #T_0_.18 (Fig 4A), and *AC2*+*AC4* stack lines #T_0_.10, #T_0_.5, #T_0_.28 and #T_0_.1 (Fig 4C and 4D).

**Table 1:** Summary of the transformation of 4-day-old cotyledonary node explants of *Vigna unguiculata* cv. Pusa Komal co-cultivated with *Agrobacterium tumefaciens* EHA105 harbouring *MYMIV RNAi-AC2* construct.

Exp. no.	No. of explants inoculated in *Agrobacterium* suspension	No of shoot recovered on selection medium	Number of plants rooted	No. of plants positive for *AC2* and *nptII* genes by PCR	Transformation efficiency^a^ (%)
1	84	48	9	3	3.57
2	73	41	10	2	2.73
3	65	52	6	3	4.61
4	52	46	5	2	3.84
5	72	47	5	2	2.77
**Total /average**	**346**^b^	**46.8**^c^	**35**	**12**^b^	**3.50**^c^

^a^ Number of plants PCR positive for *npt*II, and *AC2* per number of explants co-cultivated

^b^ Total

^c^ Average response

**Table 2:** Summary of the transformation of 4-day-old cotyledonary node explants of *Vigna unguiculata* cv. Pusa Komal co-cultivated with *Agrobacterium tumefaciens* EHA105 harbouring *MYMIV RNAi-AC4* construct.

Exp. no.	No. of explants inoculated in *Agrobacterium* suspension	No. of shoot recovered on selection medium	Number of plants rooted	No. of plants positive for *AC4* and *nptII* genes by PCR	Transformation efficiency^a^ (%)
1	67	43	12	2	2.98
2	84	37	09	2	2.38
3	54	43	07	2	3.70
4	59	44	05	2	3.38
5	78	46	09	2	2.56
**Total /average**	**342**^b^	**42.6**^b^	**42**^b^	**10**^b^	**3.00**^c^

^a^ Number of plants PCR positive for *npt*II, and *AC4* per number of explants co-cultivated

^b^ Total

^c^ Average response

### Assessment of MYMIV resistance in transgenic cowpea plants

**Fig 5:**
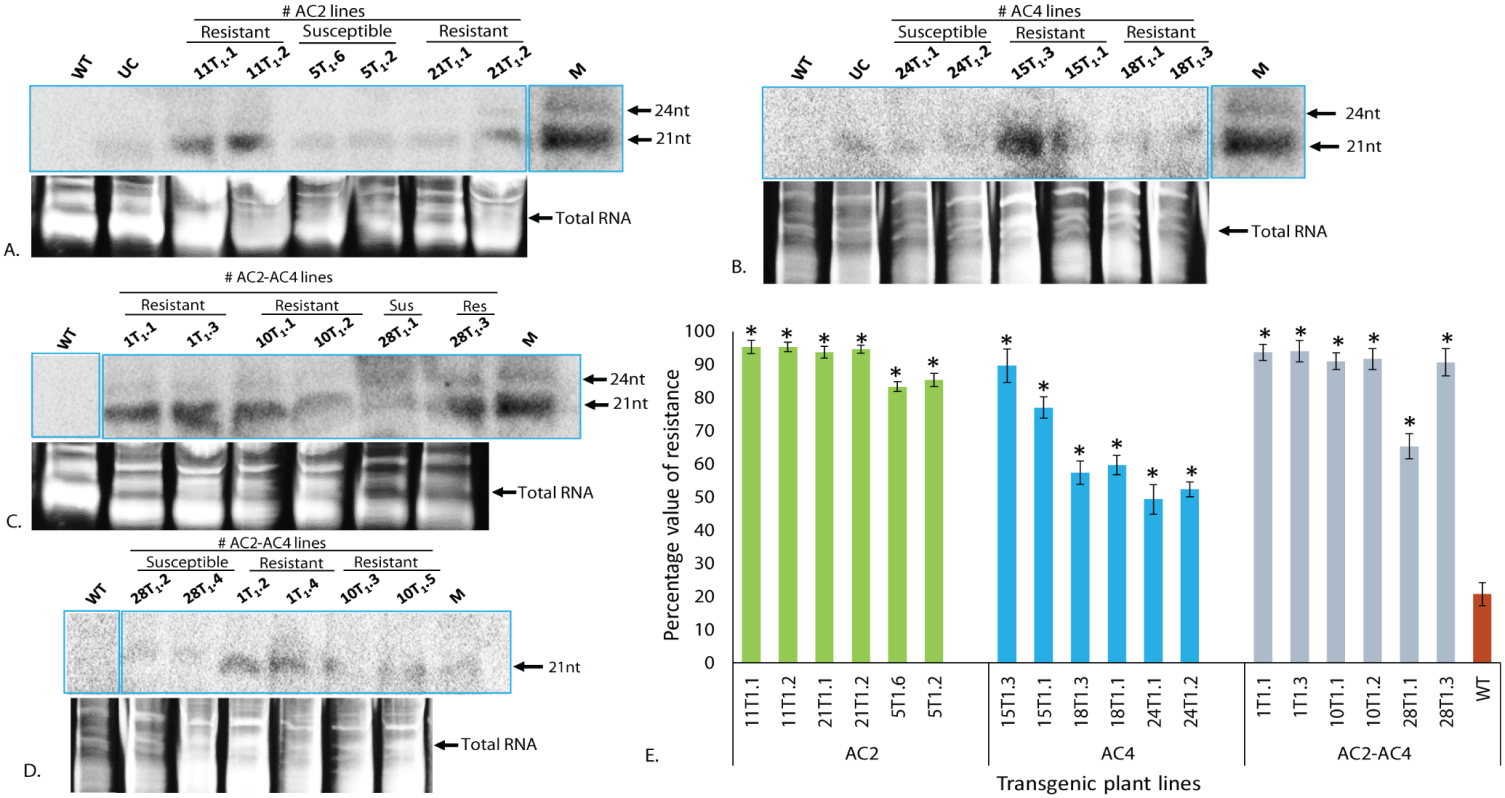
Northern blot analysis to compare siRNA production in resistant and susceptible plants in T_1_ generation derived from3 different RNAi constructs MYMIV-AC2, MYMIV-AC4 and MYMIV-AC2+AC4. Progeny plants of three independent parents of all three constructs were taken for study. **A-B.** Resistant, susceptible, unchallenged AC2 and AC4 plants are marked on the top panel. Lower panel shows the total RNA as a loading control. **C.** AC2+AC4 stacked lines were hybridized with AC2 specific probe **D.** AC2+AC4 stacked lines were hybridized with AC4 specific probe.AC2-186 nt sense and antisense transcript and AC4-197nt sense and antisense transcript were used as a probe. 21nt and 24nt indicated the siRNA markers. **E.** Graphical representation of different levels of protection obtained in transgenic cowpea lines derived from the3 RNAi constructs targeting AC2 and AC4 in the T_1_ generation after challenge with agro-infectious clones of MYMIV. Different levels of siRNA expressed by these lines positively correlate withtheir resistance levels, as shown in (A-D). The data shows the mean ± S.E of three replicate samples. *Indicates significant differences from the WT at P < 0.05.

**Fig 6:**
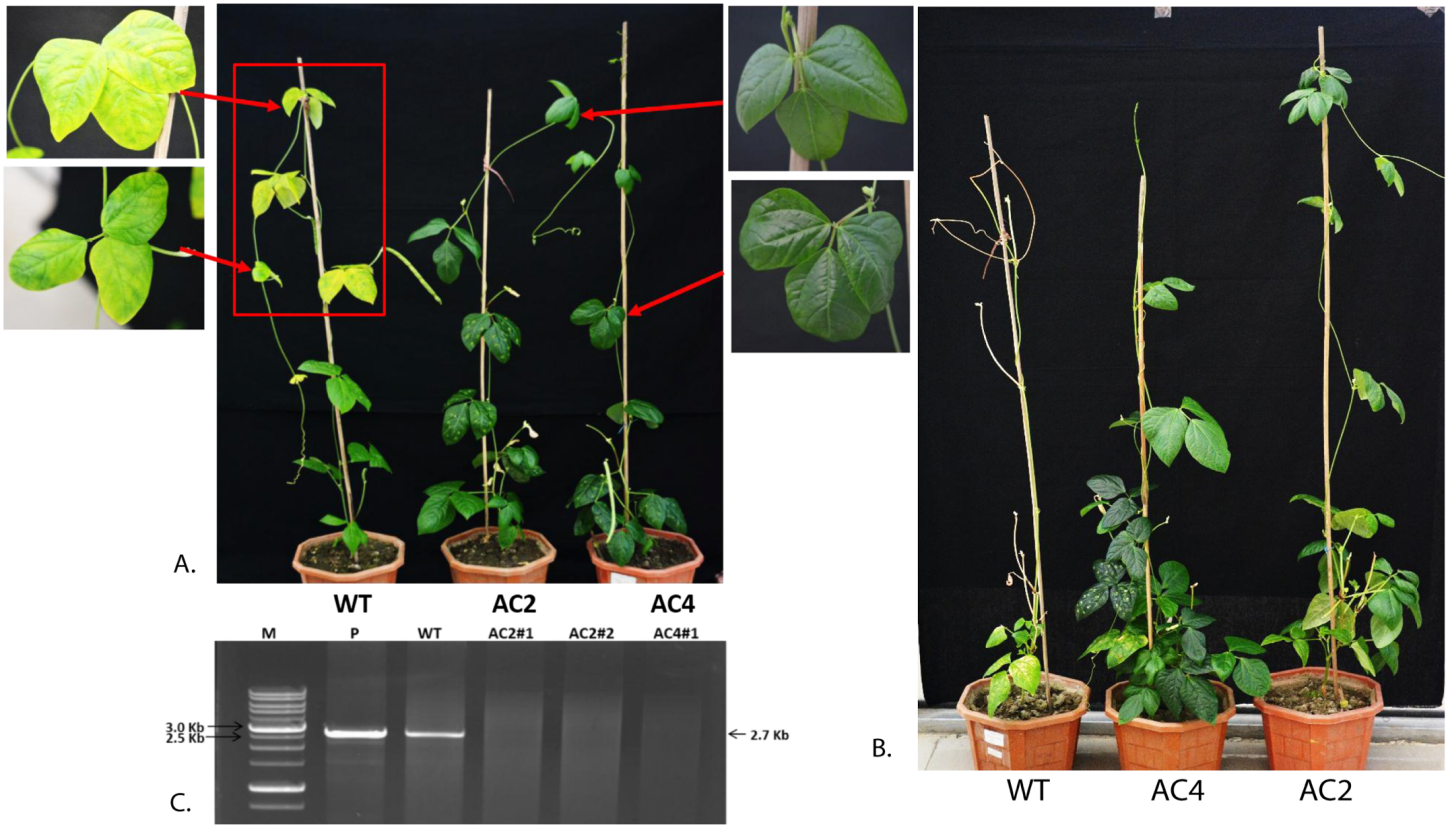
Screening of Cowpea *(Vigna unguiculata)* transgenic lines in the T_0_ generation for resistance against MYMIV and molecular confirmation of viral DNA accumulation by RCA analysis followed by restriction digestion using DNA-A specific unique cutter MfeI. **A.** Phenotype of a resistant AC2 and AC4 lines derived from the RNAi construct targeting the AC2-186 nts and AC4- 197 nts of MYMIV showing 100% protection (right) when compared with complete infection in control plants (left) when challenged with MYMIV agroinfectious viral dimers after 14^th^ day of inoculation. **B.** 9 weeks post agro infiltration. **C.** The appearance of (~2.7 kb) after digestion with MfeI of DNA-A (lane-1) indicates the presence of MYMIV infection in WT plant. Lane AC2#1, AC2#2 and AC4#1 represents the transgenic lines. Lane marked M represents 10 Kb molecular mass marker.

**Fig 7:**
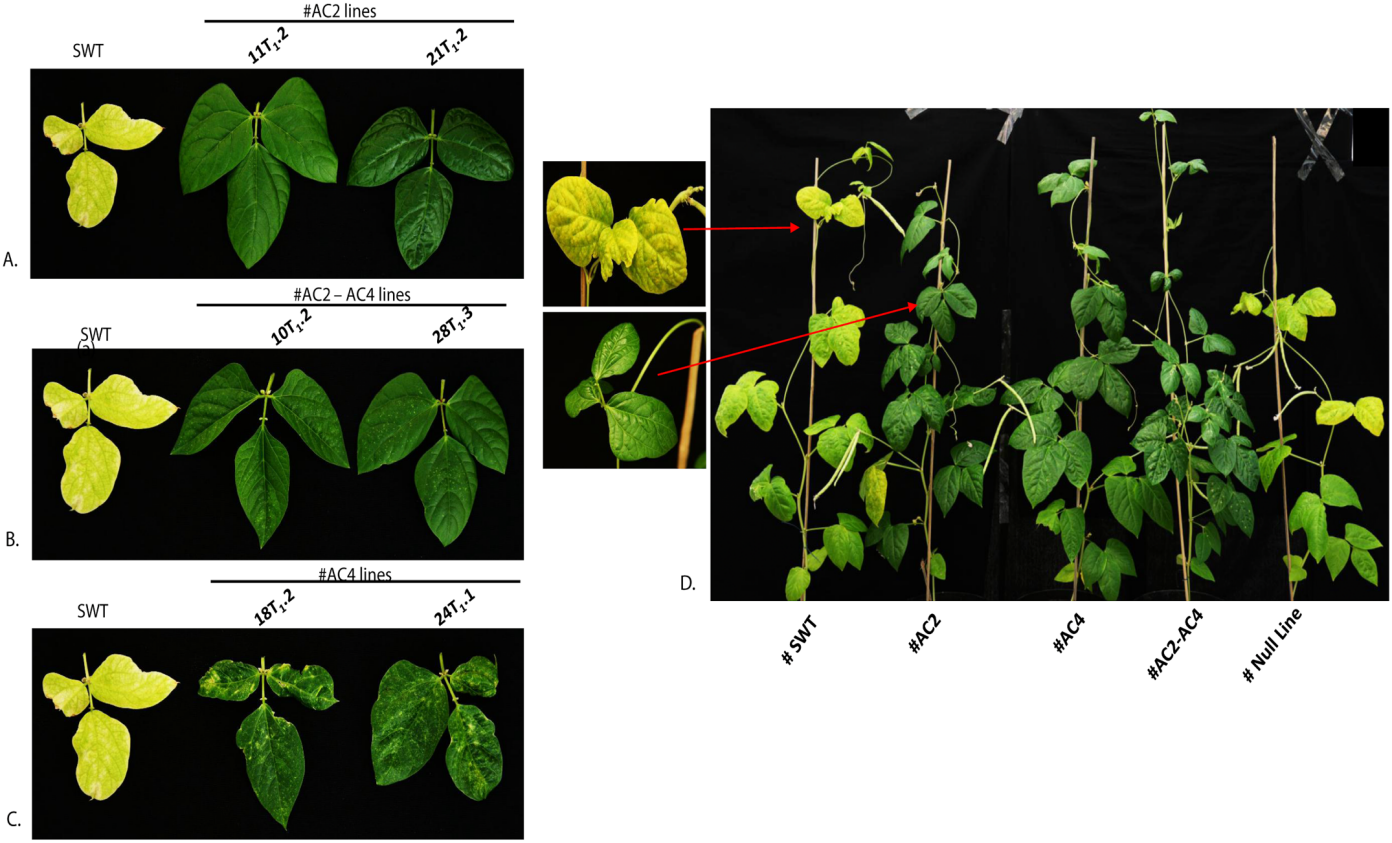
Screening of Cowpea (*Vigna unguiculata*) transgenic plant lines in the T_1_ generation for resistance against MYMIV after 5 weeks of Virus infiltration assay. **A-C.** Appearance of symptoms checked on the topmost leaf of virus challenged plants, **D.** WT and transgenic plants shown resistance after 35 days of virus challenge.

To evaluate the MYMIV resistance, the high siRNA accumulating transgenic cowpea T_0_ lines (#T_0_.5, #T_0_.11 and #T_0_.4 of *AC2*, #T_0_.15 and #T_0_.18 of *AC4*, #T_0_.1, #T_0_.10 and #T_0_.28 of *AC2*+*AC4* stack) (Fig 4) and their T_1_ segregants (Fig 5) were challenged with the agroinfectious clones of MYMIV (Figs 6 and 7; Table 3). All the untransformed WT cowpea plants and transgenic empty vector controls challenged with MYMIV developed severe symptoms after ~2 weeks of inoculation developed typical symptoms of yellow mosaic, leaf curling, severe stunting and plant necrosis (Figs 6 and 7). The RNAi-*AC2* and RNAi-*AC2+AC4* stack cowpea lines showed nearly complete resistance to MYMIV (Fig 7). Out of the three RNAi constructs evaluated, the *AC2* and *AC2*+*AC4* stack were most effective in conferring resistance to MYMIV. The two RNAi-*AC2* lines [#3 (5/5) and #7 (4/5)], and two RNAi-*AC2*+*AC4* stack lines [#7 (5/5) and #15 (4/5)] showed nearly complete resistance, with absence of any viral symptoms observed over 8 weeks (Table 3). Two RNAi-*AC4* lines (#T_0._1 and #T_0_.24) developed delayed symptoms only after 6 weeks (Table 3). Among the three different RNAi constructs tested, RNAi-*AC2* and RNAi-*AC2*+*AC4* stack generated virus resistance, with 90% of the lines demonstrating complete resistance to MYMIV as seen till T_2_ generation. Partial breakdown of resistance was observed albeit after 6 weeks in most of the RNAi-*AC4* lines with no adverse consequences on plant survival and yield. These results indicated complete MYMIV resistance in RNAi-*AC2*+*AC4* stack lines were possibly contributed predominantly by *AC2* suppression.

**Table 3:** Virus resistance assay performed for T_1_ transgenic cowpea plants challenged with agroinfectious clones of Mungbean yellow mosaic India virus (MYMIV).

Constructs	Progeny of T_0_ transgenic lines	Number of non- symptomatic plants and RCA negative/number of plants challenged	Type of symptoms
**MYMIV-AC2**	Line-1	4/5	No symptoms
	Line-3	5/5	No symptoms
	Line-7	4/5	Mild mossaic
**MYMIV- AC4**	Line-2	4/5	No symptoms
	Line-4	2/5	Mild mossaic
	Line-5	3/5	Mild mossaic
**MYMIV AC2-AC4**	Line-6	5/5	No Symptoms
	Line-15	4/5	No Symptoms
	Line-17	3/5	Mild Mossaic
**Untransformed Control**		0/5	severe symptoms in all plants

### Analysis of siRNA expression and its correlation with resistance levels

Northern blot hybridization to determine the levels of processed hpRNA specific to *AC2* and *AC4* in transgenic plants of three different RNAi constructs, before and after challenge with MYMIV showed varying levels of siRNA accumulation (Figs 4 and 5). The transgenic lines with nearly complete resistance to MYMIV were detected with higher siRNA accumulation (Figs 4A–4D and 5A–5E). The appearance of multiple forms of the transcripts, specific to *AC2* and *AC4* in Northern blots (Figs 4 and 5) was possibly due to the formation of an intermediate products of mRNA resulting from Dicer-like enzyme action during post-transcriptional gene silencing.

Conversely, some of the MYMIV-challenged transgenic plants (RNAi-*AC2* line #27, RNAi-*AC4*- line #2 and RNAi-*AC2*+*AC4*-line #33) (data not shown) or unchallenged transgenic plants (RNAi-*AC4* line #T_0_.24) were detected with lower levels of siRNAs and appearance of mild symptoms. The transgene specific siRNAs of expected sizes were detected in challenged transgenic lines (Fig 5). The level of transgene-derived siRNA was higher in RNAi-*AC2* lines #11T_1_.1, #11T_1_.2 and #21T_1_.2 as compared to line #21T_1_.1; similarly in case of RNAi-*AC2*+*AC4* stack lines, #1T_1_.1, #1T_1_.3, #10T_1_.1, #10T_1_.2, #1T_1_.2, #1T_1_.1 and #28T_1_.3 showed high accumulation of siRNA as compared to lines #28T_1_.1 and #10T_1_.3 (Fig 5). In case of RNAi-*AC4*, siRNA accumulation was significantly higher in the lines #15T_1_.3 and #15T_1_.1 except in #24T_1_.2 (Fig 5). We found a positive correlation with a correlation coefficient (*R*) of 85–90%, between the amounts of siRNA accumulation (Figs 4 and 5) in the transgenic lines (T_0_ and T_1_) and the level of MYMIV resistance exhibited by each of them, across all the RNAi constructs. The transgenic lines displaying nearly complete resistance (RNAi-*AC2* line #5, #11, #21, RNAi-*AC4* line #15, #18, RNAi-*AC2*+*AC4* line #1, #10 and #28) were found with higher accumulation of transgene-specific siRNA (Fig 4), while the plants with mild symptoms had low accumulation of siRNA. The RNAi-*AC4* transgenic lines with low accumulation of siRNA (line #18T_1_.1 and #24T_1_.2) showed low resistance to the virus. The transgenic lines derived from RNAi-*AC2* and RNAi-*AC2*+*AC4* stack were detected with high accumulation of siRNA, and consequently these lines showed nearly complete protection to MYMIV (Fig 7).

### Quantification of MYMIV DNA in virus-challenged transgenic cowpea plants and correlation with their resistance levels

**Fig 8:**
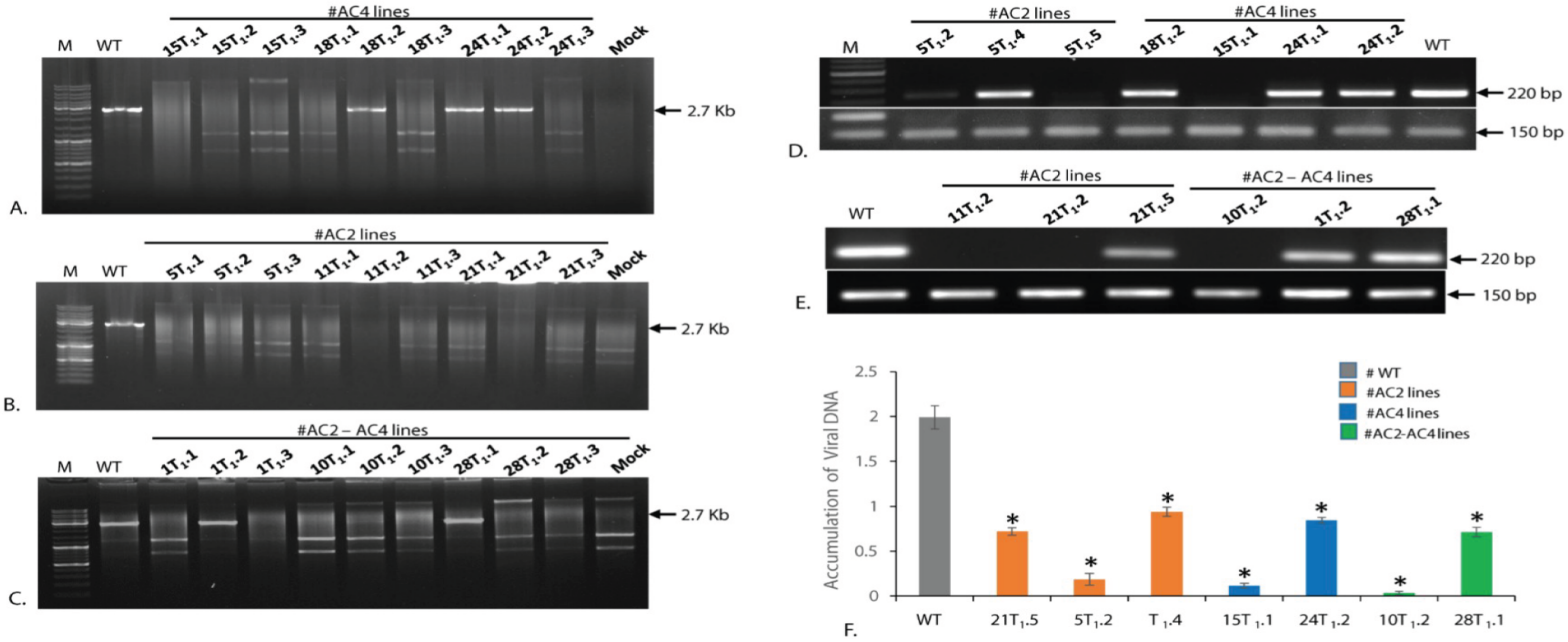
Analysis for Viral DNA accumulation and its expression in T_1_ generation transgenic cowpea lines. **A-C.** MfeI for MYMIV-DNA-A and DraI for MYMIV-DNB-B digested rolling circle amplification (RCA) products representing viral DNA in the topmost leaf at 30 days post-infiltration (dpi). WT represents the untransformed plant challenged with the agro-infectious clone of MYMIV. Mock- indicates plant challenged with null vector pCAMIA3300; sample details shown on top of the panel. M indicates 10-kb size marker; **D-E.** Expression analysis by semi quantitative RT-PCR of virus challenged (WT) and each of 3 independent transformed transgenic lines using, MYMIV-AV2 (220 bp) using pre-coat protein (AV2) specific primer. The 150 bp amplification of *Vu- Ubiquitin* were used as an internal control. Sample name marked on top of the panel. **F.** Quantification of virus in transgenics and WT by quantitative polymerase chain reaction (qPCR). The bar graph showing virus accumulation in different T_1_ transgenic lines [MYMIV-AC2, MYMIV-AC4 and MYMIV-AC2+AC4] at 30 days post-infiltration (dpi), calculated by real-time PCR, using coat protein primers and *Vu-Ubiquitn* as an internal control. WT represents wild-type plant inoculated with virus. The upper leaf from the bottom was taken for the study. Standard deviations are shown in the form of bars. The data shows the mean ± S.E of three replicate samples. *Indicates significant differences from the WT at P < 0.05.

After five weeks of agro-infection with MYMIV, all the resistant and susceptible transgenic cowpea lines were analyzed for viral DNA accumulation by RCA, semi-quantitative RT-PCR and qRT-PCR using primers specific to pre-coat protein (*AV2*). Viral DNA was not detected in resistant lines (RNAi-*AC2* and RNAi-*AC2*+*AC4* stack) and these lines were free from viral symptoms. However, viral DNA accumulation detected in symptomatic plants were mostly *AC4* lines while the virus challenged WT plants showed high levels of viral DNA accumulation. The level of viral DNA relative to the internal *Vu-Ubiquitin* standard was calculated in the virus challenged plants following real-time PCR after 5 weeks of virus inoculation. The Δ*Ct* value for each real-time PCR was derived by taking the values of the internal standard *Vu-Ubiquitin*, and the values obtained were considered for the calculation of the relative levels of viral DNA as shown in Fig 8F. The transgenic plant lines displaying nearly complete resistance showed absence of viral DNA. The RNAi-*AC2* lines (#5T_1_.2), RNAi-*AC4* line (#15T_1_.1), and RNAi-*AC2*+*AC4* line (#10T_1_.2) exhibiting less viral DNA accumulation with corresponding high level of siRNA accumulation showed nearly complete resistance to MYMIV. On the contrary, the RNAi-*AC2* line (#T_1_.4), RNAi-*AC4* line (24T_1_.2) and RNAi-*AC2*+*AC4* stack line (#28T_1_.1) detected with high viral DNA accumulation (around 2-fold less than the WT plant) (Fig 8D–8F) had a significantly low siRNA accumulation and appearance of disease symptoms. In all cases, we found an inverse relationship between siRNA level and viral DNA accumulation.

### Yield of MYMIV-resistant transgenic cowpea lines

The plant height, branch number, pod number/plant, seed number/plant, seed weight/plant, 100 seed weight and 10 seed length, were analyzed in T_1_ transgenic cowpea lines under greenhouse conditions to investigate effects of high level of siRNA accumulation with concomitant MYMIV resistance on agronomic and yield traits. The virus resistant transgenic lines (#5, #11 and #21 of RNAi-*AC2*; #18 and #24 of RNAi-*AC4*; #1, #10 and #28 of RNAi-*AC2*+*AC4* stack) showed similar seed yield with normal phenotype that of unchallenged WT cowpea plants (S4 Table). No significant difference in seed traits was observed among the transgenic MYMIV-resistant cowpea plants.

## Discussion

Yellow mosaic disease (YMD) caused by the bipartite begomovirus, MYMIV is a serious impediment to production of most of the grain legumes in the Indian subcontinent [14, 51–53]. Among the various biotechnological approaches available, RNAi has been most successful in controlling plant viral diseases including those caused by geminiviruses [32, 54–55]. RNAi or post-transcriptional gene silencing (PTGS) is triggered by the formation of double-stranded RNA (dsRNA) that are cleaved to form siRNAs by DCLs and RISC [24, 56–57]. Therefore, the hairpin RNAi constructs that direct expression of dsRNA transcripts efficiently induce targeted gene silencing due to the sequence specific degradation of target RNA by siRNAs [58].

Application of RNAi for trait improvement in cowpea depends on availability of reliable transformation system. Cowpea is highly recalcitrant to genetic transformation and till date, only a few laboratories have succeeded in transforming cowpea [59]. In the past, we have established efficient *Agrobacterium-*mediated transformation of cowpea by improving transformation efficiency through the use of extra copies of vir genes [60], sonication and vacuum infiltration [61], seedling preconditioning in thidiazuron [62] and by employing positive selection [63]. Previously we have successfully developed transgenic cowpea resistance to storage pest [64], field insects [65] and tolerance to salinity [43, 66].

The sequences conserved within the *AC2* and *AC4* ORFs of seven different cowpea begomoviruses isolates in India were chosen as the RNAi targets [67–69]. Some of these cowpea begomovirus isolates are also known to co-infect cowpea impacting synergistically on the disease severity [70]. The rationale behind selecting the conserved regions (186 bp of *AC2* and 197 bp of *AC4*) of seven begomoviruses cowpea isolates for development of RNAi constructs was to confer broad spectrum resistance to cowpea infecting begomoviruses.

In this study, we generated 27 transgenic cowpea lines of RNAi-*AC2*, 34 plant lines of RNAi-*AC4* and 36 plant lines of RNAi-*AC2*+*AC4* stack constructs through *Agrobacterium*-mediated transformation with a transformation efficiency of 3.5%. All the transgenic plants were phenotypically similar to that of WT cowpea plants, indicating the absence of any siRNA off-targets in greenhouse conditions. The mechanism of *Agrobacterium*-mediated plant transformation is not completely understood, and the integration of T-DNA into the host genome is believed to be a random process [71–72]. For instance, the *AC2* T_1_ transgenic line, #21T_1_.1 expressed very low levels of siRNA, while one of its sibling #21T_1_.2 showed high level of siRNA accumulation concomitant with nearly complete virus resistance (Fig 5A). The T_0_ RNAi-*AC2* and RNAi-*AC4* cowpea lines challenged with MYMIV (RNAi-*AC2* line #T_0_.5, #T_0_.11, #T_0_.21, and RNAi-*AC4*, line #T_0_.15 and #T_0_.18) showed nearly complete resistance to the virus concomitant with high level of siRNA accumulation (Fig 4A–4C) and absence of viral DNA in the challenged plants. The RNAi-*AC4* line #T_0_.3 and #T_0_.21 that developed mild mosaic symptoms (after 35 days of virus inoculation) had low level of siRNA accumulation. Some of the RNAi-*AC4* lines such as #T_0_.21 showed recovery from the virus infection at the later stage (after 55 days of virus inoculation).

Recent studies in tobacco have shown RNAi mediated targeting of *AC2* of Mungbean yellow mosaic virus (MYMV) efficiently blocked the accumulation of viral DNA [73]. Suppression of the *AC4*, which acts as a gene-silencing suppressor, through expression of *AC4* hpRNA has generated effective resistance against different begomoviruses [74–76]. A recent study has shown that *AC2* as a better target than *AC4* for RNAi-based control of African cassava mosaic virus [22]. Other RNAi studies for resistance to tomato chlorotic mottle virus (TCMV) [77] and Pepper golden mosaic virus (PGMV) [78] indicated *AC2* targeting was very effective in controlling TCMV and PGMV. High resistance to ACMV and ToLCTWV in both transient and stable transgenic *Nicotiana benthamiana*, generated through RNAi targeting of viral *AC2*/*C2* strongly suggested *AC2* of either bipartite or monopartite begomoviruses an effective target for developing virus resistance [79].

Our studies also show that there is a positive correlation between the levels of siRNA accumulation to that of extent of resistance to MYMIV. Further, the MYMIV resistant transgenic cowpea lines did not support the replication and proliferation of MYMIV. This is in complete agreement with previous reports on positive correlation among the levels of siRNA accumulation, extent of resistance to cassava viruses and levels of viral DNA accumulation [22]. Thus the level of siRNA accumulation is a key determinant for the extent of virus resistance and this could be used as an indicator to identify the best performing transgenic plant line/s. We observed high level of siRNA accumulation concomitant with high resistance to MYMIV in most of the transgenic lines consistently over two successive generations and further evaluation of few more generations could answer the durability of introduced trait. Absence of any yield penalty in MYMIV-resistant RNAi-transgenic cowpea plants indicated that the RNAi suppression of *AC2* and *AC4* of MYMIV possibly had no off targets in greenhouse growth conditions. Our studies clearly demonstrate that the RNAi targeting of *AC2* and *AC4* imparted nearly complete resistance to MYMIV in cowpea with no yield penalty. This is the first report on the development of transgenic cowpea plants resistant to a geminivirus. MYMIV is known infects several legumes in India [7], and therefore our strategy could be implemented to develop MYMIV resistance in other important legumes of Indian subcontinent including mungbean and soybean. This study encourages the use of RNAi-technology for the effective control of plant viruses in diverse crop plants without risking negative effect on the ideotype of the crop plant.

## Supporting information

S1 Table AC2 and AC4 target sequences used for preparation of RNAi constructs against seven begomoviruses cowpea isolates. (DOCX)

S2 Table Sequences of primers used for the study. (DOCX)

S3 Table PCR conditions used for analysis. (DOCX)

S4 Table Agronomic characteristics of WT and virus challenge transgenic cowpea lines in T_1_ generation. (DOCX)

S1 Fig **A. Virus infected cowpea plants in field at diverse locations of India**, Jharkhand, Karnataka, Maharashtra and Chattishgarh (Left panel) **B.** Cowpea leaves collected from field of various locations: a-c Chhattisgarh, d-f Assam, g-j Jharkhand k-n, Maharashtra (right panel) **C.** Rolling circle amplification followed by restriction digestion of healthy and infected samples (1–4). Lanes A, B, C, D and E represent digestion with *EcoR*I, *EcoR*V, *Hind*III, *BamH*I, *Sac*I respectively. The appearance of 2.7 kb and 1.3 kb bands on digestion with above enzymes indicates the presence of begomovirus. Lane marked M represents the molecularmass marker Lamda DNA *EcoR*I+ *Hind*III. Lane marked P represents the positive control (pUC19 RCA kit supplied).
(TIF)
