# Supplementary material for: RNAi-derived transgenic resistance to Mungbean yellow mosaic India virus in cowpea
Source: PLoS One. 2017 Oct 27;12(10):e0186786. doi: 10.1371/journal.pone.0186786 (PMC5659608; doi:10.1371/journal.pone.0186786)
Supplement: S1 Table — (DOCX) [file pone.0186786.s001.docx]

**S1 Table** AC2 and AC4 target sequences used for preparation of RNAi constructs against seven begomoviruses cowpea isolates

1. **Gene AC2 transcription activator protein (397 nt)**

ATGCGGAGTTCTACACCCTCAAGGAACCATTGTTCTCCTCCGTCAATCAAGGTTCAACACAGGGTCGCCAAGAAGAAAGCAATTCGACGATCTCGAATTGACTTGAAATGTGGGTGTAGCTATTACATCCATATCAACTGCCGTAACTATGGATTTTCGCACAGGGGAGAGCATCACTGCAGCTCAACTCAGGAATGGCGTCTATATCTGGGAGGTGCAAAATCCCCTCTCTTTCAAGATCATGCACCACAGTCCAATTCAAGCAGGGTCCAGAATGATTGTCACCAAGCTACGCATAATGTTCAACCACGGGTTGAAGAAAGCACTGGGGATGCACAAGTGCTTCCTGGACTTGAAGATCTACCATTATATGAAGGCGACTTCTGGGATGATCTTA

1. **Gene AC1 replication initiator protein (805 nt)**

ATGCCAAGGGATGGTCGTTTTGCAATAAACGCAAGAAACTATTTCTTGACATATCCCAAATGTCCTCTTACAAAGGAGGAAGCTCTTGAACAGCTTCTCGCATTATCAACACCTGTTAACAAGAAATTCATTCGCATCTGTCGCGAACTTCATGAAGATGGACAGCCTCATCTCCATGTTCTGCTTCAGTTCGAAGGGAAACAACAAACGAGGAACCAAAGGTTCTTCGACCTCTATTCCAGATGCAGATCGGCACATTACCATCCGAACATTCAGGCAGCTAAAAGCTGCTCAGACGTTAAAAAATACATGGAGAAAGACGGAGACGTCCTTGATCATGGAACTTTCCAAATCGATGGCCGATCAGCTCGAGGAGGTAAACAATCTGCCAACGACGCATACGCCGAGGCACTCAATTGTGGATCGAAATTGGAGGCCCTCCTTATATTAAAAGAAAAGGCTCCTAAGGATTTTATTTTACAATTTCATAATTTGAATTGTAATTTGTCTCGTATTTTCTCAGAGCCTGTCCAGGCATATGAGTCGCCTTTTACGATGGAGTCATTTAACAAGGTTCCGAGCTACATTTCTTCATGGGCTGAAAGAAATGTGAGGGATCCCGCTGCGCGGCCGGAGAGACCTATTAGTATTGTTATTGAGGGAGATAGTCGCACGGGTAAAACCATGTGGGCACGTGCCATAGGTTCTCATAATTATCTTTGCGGCCATTTGGATCTAAACGACAAAACATACTCTAACGAGGCATGGTACAACGTCATTGATGACGTTGATCCACATTATTGAA

1. **Gene AC4 (297 nt)**

AAGATGGACAGCCTCATCTCCATGTTCTGCTTCAGTTCGAAGGGAAACAACAAACGAGGAACCAAAGGTTCTTCGACCTCTATTCCAGATGCAGATCGGCACATTACCATCCGAACATTCAGGCAGCTAAAAGCTGCTCAGACGTTAAAAAATACATGGAGAAAGACGGAGACGTCCTTGATCATGGAACTTTCCAAATCGATGGCCGATCAGCTCGAGGAGGTAAACAATCTGCCAACGACGCATACGCCGAGGCACTCAATTGTGGATCGAAATTGGAGGCCCTCCTTATATTAA
