# Supplementary material for: RNAi-derived transgenic resistance to Mungbean yellow mosaic India virus in cowpea
Source: PLoS One. 2017 Oct 27;12(10):e0186786. doi: 10.1371/journal.pone.0186786 (PMC5659608; doi:10.1371/journal.pone.0186786)
Supplement: S2 Table — (DOCX) [file pone.0186786.s002.docx]

**S2 Table** Sequences of primers used for the study

| **Name and purpose** | | | | | **Primer name** | **Sequence(5’→3’)** |
| --- | --- | --- | --- | --- | --- | --- |
| Cloning of AC2 in sense orientation | | | | | AC2 S Fw | TATATT**CTCGA**GGCAATTCGACGATCTC |
|  |  |  |  |  | AC2 S Rv | TATTAA**GGTACC**AATTGGACTGTGGTG |
| Cloning of AC2 in antisense orientation | | | | | AC2 AS Fw | TATACC**TCTAGA**GCAATTCGACGATCTC |
|  |  |  |  |  | AC2 AS Rv | TATTGG**ATCGAT**AATTGGACTGTGGTG |
| Cloning of AC4 in sense orientation | | | | | AC4 S Fw | TATATT**CTCGAG**AAGATGGACAGCCTCA |
|  |  |  |  |  | AC4 S Rv | TAGGAA**GGTACC**TTGGAAAGTTCCATGA |
| Cloning of AC4 in antisense orientation | | | | | AC4 AS Fw | TAGGAA**TCTAGA**TTGGAAAGTTCCATGA |
|  |  |  |  |  | AC4 AS Rv | TCCTGG**ATCGAT**TTGGAAAGTTCCATGA |
| Cloning of AC2 in sense for AC2-AC4 construct | | | | | *AC2* S2 Fw | ATGCTT**CTCGA**G**TT**GCAATTCGACGATCTC |
|  |  |  |  |  | *AC2* S2 Rv | TAGTAA**GAATTCTT**AATTGGACTGTGGTG |
| Cloning of AC4 in sense for AC2-AC4 construct | | | | | *AC4* S2 Fw | TATATT**GAATTC**AAGATGGACAGCCTCA |
|  |  |  |  |  | *AC4* S2 Rv | GCGCTT**GGTACC**TTGGAAAGTTCCATGA |
| Cloning of AC2 in antisense for AC2-AC4 construct | | | | | *AC2* AS2 Fw | TAATAA**TCTAGATT**GCAATTCGACGATCTC |
|  |  |  |  |  | *AC2* AS2 Rv | TACTGG**GGATCCTT**AATTGGACTGTGGTG |
| Cloning of AC4 in antisense for AC2-AC4 construct | | | | | *AC4* AS2 Fw | TAATGG**GGATCC**AAGATGGACAGCCTCA |
|  |  |  |  |  | *AC4* AS2 Rv | ACAGAA**ATCGAT**TTGGAAAGTTCCATGA |
| MYMIV-AC2 for screening of transgenic plants | | | | | *AC2*Fw | TTCGACGATCTCGAATTGACTTGAAAT |
|  |  |  |  |  | *AC2*Rv | AATTGGACTGTGGTGCATGATCTTGA |
| MYMIV-AC4 for screening of transgenic plants | | | | | *AC4*Fw | GCAATTCGACGATCTCGAATTGAC |
|  |  |  |  |  | *AC4*Rv | TTGGAAAGTTCCATGATCAAGGAC |
| Real Time PCR | | | | | *Vu-Ubiquitin*Fw | TCAGTTGAGGCCGAAGAAGA |
|  |  |  |  |  | *Vu-Ubiquiti*n Rv | AAACCAGTCCCAGTCCCAAA |
|  |  |  |  |  | *AV2*-Fw | TAT ACA GTC GGT AAA ACC GAG GTT |
|  |  |  |  |  | *AV2*Rv | CTA ATT CTC GTG GTT TTATGT ACC |
| NptII for probe prep. and transgenic plant screening | | | | | *NptII*Fw | GTGGAGAGGCTATTCGGCTA |
|  |  |  |  |  | *Npt*IIRv | CCACCATGATATTCGGCAAC |
| MYMIV -AC2 size markers for northern | | | | | *AC2*-21 nt | GCAATTCGACGATCTCGAATT |
|  |  |  |  |  | AC2-24 nt | GCAATTCGACGATCTCGAATTGAC |
| MYMIV -AC4 size markers for northern | | | | | *AC4*-21 nt | AAGATGGACAGCCTCATCTCC |
|  |  |  |  |  | *AC4*-24 nt | AAGATGGACAGCCTCATCTCCATG |
| M13 for cloning confirmation in pGEMT-EASY vector | | | | | M13 Fw | GTAAAACGACGGCCAG |
|  |  |  |  |  | M13 Rv | CAGGAAACAGCTATGAC |
