# Supplementary material for: RNAi-derived transgenic resistance to Mungbean yellow mosaic India virus in cowpea
Source: PLoS One. 2017 Oct 27;12(10):e0186786. doi: 10.1371/journal.pone.0186786 (PMC5659608; doi:10.1371/journal.pone.0186786)
Supplement: S3 Table — (DOCX) [file pone.0186786.s003.docx]

**S3 Table** PCR conditions used for analysis

|  |  |  |  |  |  |  |
| --- | --- | --- | --- | --- | --- | --- |
| **Name of primer** | **Primary denaturation** | **Secondary denaturation** | **Primer annealing** | **Initial extension** | **Final extension** | **No. of cycle repeat** |
| **NptII** | 95°C/ 4 min. | 95°C/ 1 min. | 58°C/ 30sec. | 72°C/ 1 min. | 72°C/ 10 min. | 35 |
| **AC2** | 95°C/ 4 min. | 95°C/ 1 min. | 60°C/ 45sec. | 72°C/ 1 min. | 72°C/ 10 min. | 35 |
| **AC4** | 95°C/ 4 min. | 95°C/ 1 min. | 58°C/ 45sec. | 72°C/ 1 min. | 72°C/ 10 min. | 35 |
| **Vu-Ubiquitin** | 95°C/ 4 min. | 95°C/ 30 sec. | 59°C/ 45sec. | 72°C/ 1 min. | 72°C/ 5 min. | 35 |
| **AV2** | 95°C/ 4 min. | 95°C/ 30 sec. | 60°C/ 45sec. | 72°C/ 1 min. | 72°C/ 5 min. | 35 |
