# Supplementary material for: RNAi-derived transgenic resistance to Mungbean yellow mosaic India virus in cowpea
Source: PLoS One. 2017 Oct 27;12(10):e0186786. doi: 10.1371/journal.pone.0186786 (PMC5659608; doi:10.1371/journal.pone.0186786)
Supplement: S4 Table — (DOCX) [file pone.0186786.s004.docx]

**S4 Table** Agronomic characteristics of WT and virus challenge transgenic cowpea lines in T_1_ generation.

|  |  |  | #Control** | | | | | #AC2* | | | | | | | | | | | | #AC4* | | | | | | | | | | | | #AC2-AC4* | | | | | | | | | | | |  |
| --- | --- | --- | --- | --- | --- | --- | --- | --- | --- | --- | --- | --- | --- | --- | --- | --- | --- | --- | --- | --- | --- | --- | --- | --- | --- | --- | --- | --- | --- | --- | --- | --- | --- | --- | --- | --- | --- | --- | --- | --- | --- | --- | --- | --- |
| **Parameters** | | **UWT** | | | **SWT** | | | **Line #5** | | | | **Line #11** | | | | **Line #21** | | | | **Line#15** | | | | **Line 18** | | | | **Line 24** | | | | **Line #1** | | | | **Line 10** | | | | **Line #28** | | | |  |
| **Plant height (cm)** | | 93.3 ± 1.16 | | | 42 ± 0.15 | | | 95.3 ± 1.66 | | | | 125.3 ± 0.90 | | | | 105 ± 1.15 | | | | 79.3 ± 2.72 | | | | 94.3 ± 2.17 | | | | 115 ± 1.52 | | | | 105.3 ± 1.76 | | | | 103.3 ± 0.90 | | | | 115 ± 1.15 | | | |  |
| **Branch number** | | 12.6 ± 1.08 | | | 7.3 ± 0.88 | | | 13.6 ± 0.88 | | | | 15.6 ± 0.33 | | | | 14 ± 1.15 | | | | 12 ± 1.52 | | | | 13 ± 1.12 | | | | 14.3 ± 0.68 | | | | 12.9 ± 0.44 | | | | 13 ± 0.53 | | | | 13.6 ± 0.65 | | | |  |
| **Pod number/Plant** | | 08 ± 0.45 | | | 02 ± 0.47 | | | 09 ± 0.52 | | | | 11 ± 0.48 | | | | 10.2 ± 0.68 | | | | 6.3 ± 0.88 | | | | 07 ± 0.47 | | | | 09.6 ± 0.18 | | | | 07 ± 0.42 | | | | 09 ± 0.48 | | | | 08.2 ± 0.18 | | | |  |
| **Seed number/Plant** | | 87 ± 3.21 | | | 15.6 ± 2.02 | | | 107 ± 2.21 | | | | 110.3 ± 5.7 | | | | 97.3 ± 7.21 | | | | 74 ± 4.16 | | | | 91.3 ± 3.28 | | | | 107 ± 4.21 | | | | 87 ± 1.29 | | | | 113.3 ± 4.5 | | | | 99.3 ±2.21 | | | |  |
| **Seed weight (g)/Plant** | | 150.7 ± 13.32 | | | 17.34 ± 4.78 | | | 160.7 ± 14.32 | | | | 164.7 ± 19.4 | | | | 141.7 ± 18.4 | | | | 111.3 ± 19.1 | | | | 138.3 ± 12.07 | | | | 165 ± 14.1 | | | | 130.7 ± 14.32 | | | | 174.7 ± 19.4 | | | | 148.7 ± 14.4 | | | |  |
| **10 Seed weight (mg)** | | 1239 ± 4.39 | | | 902.1 ± 3.78 | | | 1209 ± 5.19 | | | | 1257 ± 6.80 | | | | 1341.6 ± 4.93 | | | | 1228.6 ± 7.68 | | | | 1324 ± 7.36 | | | | 1352.6 ± 5.06 | | | | 1219 ± 5.19 | | | | 1197 ± 6.80 | | | | 1341.6 ± 4.93 | | | |  |
| **10 Seed length (cm)** | | 7.7 ± 0.52 | | | 6.3 ± 0.11 | | | 7.6 ± 0.88 | | | | 8.2 ± 0.08 | | | | 7.9 ± 0.11 | | | | 7.8 ± 0.05 | | | | 7.4 ± 0.17 | | | | 7.9 ± 0.14 | | | | 7.9 ± 0.88 | | | | 8.1 ± 0.18 | | | | 7.4 ± 0.11 | | | |  |
|  |  |  |  |  | |  |  | |  | |  | |  | |  | |  | |  | |  | |  | |  | |  | |  | |  | |  | |  | |  | |  | |  | |  | |
| * Average of five plants from each parent | | | | | | | |  | |  | |  | |  | |  | |  | |  | |  | |  | |  | |  | |  | |  | |  | |  | |  | |  | |  | |  |
| ** Average of five challanged/non-challanged control plants | | | | | | | | | | | |  | |  | |  | |  | |  | |  | |  | |  | |  | |  | |  | |  | |  | |  | |  | |  | |  |
