# Supplementary figures and images for: RNAi-derived transgenic resistance to Mungbean yellow mosaic India virus in cowpea
Source: PLoS One. 2017 Oct 27;12(10):e0186786. doi: 10.1371/journal.pone.0186786 (PMC5659608; doi:10.1371/journal.pone.0186786)

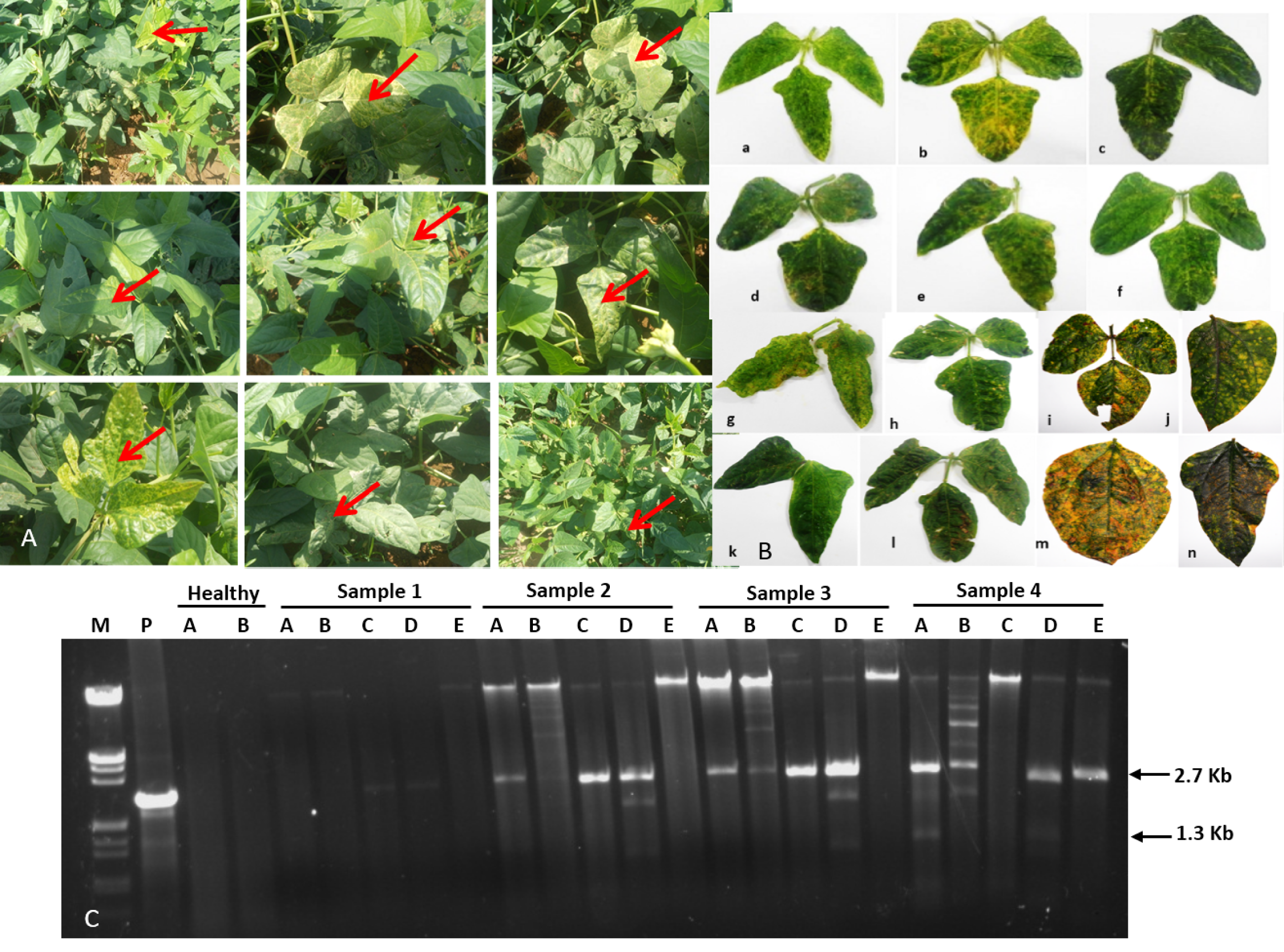

Supplement: S1 Fig — A. Virus infected cowpea plants in field at diverse locations of India, Jharkhand, Karnataka, Maharashtra and Chattishgarh (Left panel) B. Cowpea leaves collected from field of various locations: a-c Chhattisgarh, d-f Assam, g-j Jharkhand k-n, Maharashtra (right panel) C. Rolling circle amplification followed by restriction digestion of healthy and infected samples (1–4). Lanes A, B, C, D and E represent digestion with EcoRI, EcoRV, HindIII, BamHI, SacI respectively. The appearance of 2.7 kb and 1.3 kb bands on digestion with above enzymes indicates the presence of begomovirus. Lane marked M represents the molecularmass marker Lamda DNA EcoRI+ HindIII. Lane marked P represents the positive control (pUC19 RCA kit supplied). (TIF) [file pone.0186786.s005.tif]
